# Supplementary material for: A high-resolution mRNA expression time course of embryonic development in zebrafish
Source: eLife. 2017 Nov 16;6:e30860. doi: 10.7554/eLife.30860 (PMC5690287; doi:10.7554/eLife.30860)
Supplement: Supplementary file 6. [file elife-30860-supp6.zip › biolayout-clusters-files/Cluster094-genes.html]

Cluster094


# Cluster094: Genes

| Ensembl ID | Gene Name | Chr | Start | End | Biotype |
| --- | --- | --- | --- | --- | --- |
| ENSDARG00000026444 | apoob | 24 | 21833056 | 21844232 | protein\_coding |
| ENSDARG00000087693 | atox1 | 14 | 25685717 | 25688517 | protein\_coding |
| ENSDARG00000037860 | cox6b2 | 16 | 12614027 | 12621691 | protein\_coding |
| ENSDARG00000095273 | cox8a | 21 | 28459499 | 28464009 | protein\_coding |
| ENSDARG00000062752 | mfap3l | 7 | 7428826 | 7442817 | protein\_coding |
| ENSDARG00000060489 | mrpl32 | 16 | 43441392 | 43443206 | protein\_coding |
| ENSDARG00000045296 | myeov2 | 24 | 26139473 | 26157334 | protein\_coding |
| ENSDARG00000013333 | ndufa10 | 9 | 23929637 | 23936411 | protein\_coding |
| ENSDARG00000014915 | ndufab1b | 3 | 35405613 | 35411902 | protein\_coding |
| ENSDARG00000025549 | ndufaf1 | 13 | 33115700 | 33121910 | protein\_coding |
| ENSDARG00000041314 | ndufb9 | 16 | 6446261 | 6450601 | protein\_coding |
| ENSDARG00000006290 | ndufs5 | 19 | 35770076 | 35774436 | protein\_coding |
| ENSDARG00000095921 | si:ch1073-325m22.2 | 2 | 57055559 | 57069993 | processed\_transcript |
| ENSDARG00000045364 | trappc3 | 19 | 36135782 | 36146281 | protein\_coding |
